# Interaction and joint effect of ALT and chronic liver disease on liver cancer in type 2 diabetes patients

**DOI:** 10.18632/oncotarget.21804

**Published:** 2017-10-10

**Authors:** Tsai-Chung Li, Chia-Ing Li, Chiu-Shong Liu, Pao-Hsuan Lin, Wen-Yuan Lin, Chih-Hsueh Lin, Sing-Yu Yang, Jen-Huai Chiang, Cheng-Chieh Lin

**Affiliations:** ^1^ Department of Public Health, College of Public Health, China Medical University, Taichung, Taiwan; ^2^ Department of Healthcare Administration, College of Medical and Health Science, Asia University, Taichung, Taiwan; ^3^ School of Medicine, College of Medicine, China Medical University, Taichung, Taiwan; ^4^ Department of Medical Research, China Medical University Hospital, Taichung, Taiwan; ^5^ Department of Family Medicine, China Medical University Hospital, Taichung, Taiwan; ^6^ Management Office for Health Data, China Medical University Hospital, Taichung, Taiwan

**Keywords:** alanine transaminase, cohort study, hepatocellular carcinoma, type 2 diabetes

## Abstract

**Background:**

This study examined whether serum alanine transaminase (ALT) and chronic liver diseases were interactively, jointly, or independently associated with hepatocellular carcinoma (HCC) risk in type 2 diabetic patients.

**Materials and Methods:**

A retrospective cohort study was conducted in 46,369 Chinese type 2 diabetic patients, aged 30 and older, in National Diabetes Care Management Program in 2002-2004. These data were analyzed by multivariate Cox proportional hazards models.

**Results:**

Mean follow-up period was 8.20 years. Multivariate-adjusted hazard ratios of HCC were 2.85 (95% confidence interval, CI: 2.45–3.31), 3.80 (3.04–4.76), and 3.89 (3.08–4.91) for patients with a level of ALT 40–80, 80–120, and >120 U/L, respectively, compared with patients with a level of ALT < 40 U/L after multivariable adjustment. Significant hazard ratios of HCC for patients with a level of ALT ≥ 40 U/L and alcoholic liver damage, nonalcoholic fatty liver disease, liver cirrhosis, hepatitis B virus and hepatitis C virus infection, or any one of these chronic liver diseases compared with patients with ALT level < 40 U/L and no counterpart comorbidity were observed. Significant effect modifications were observed between ALT level with liver cirrhosis and HBV.

**Conclusions:**

Results suggest significant effect modification and joint associations of ALT ≥ 40 U/L and chronic liver diseases. Diabetes care should provide lifestyle or treatment interventions to manage ALT level, liver cirrhosis and hepatitis B virus infection for reducing burden of HCC.

## INTRODUCTION

In 2012, hepatocellular carcinoma (HCC) was the third leading cause of cancer mortality worldwide. The incidence of HCC was ranked as the seventh most common cancer worldwide, and its incidence rate was approximately 11.1 per 100,000 persons in 2012 [1]. International Agency for Research on Cancer reported that five-year prevalence is 12.2 per 100,000 persons (633,170 cases) [1]. In Taiwan, HCC is the second leading cause of cancer death [2]. According to Ministry of Health and Welfare in Taiwan, age-standardized mortality of HCC was 22.8 per 100,000 persons in 2015 [2]. Moreover, age-standardized incidence of HCC was 35.0 per 100,000 persons in 2012, whereas mortality and incidence are approximately threefold higher than global occurrences [1, 3].

Type 2 diabetes has rapidly become prevalent globally. World Health Organization reports that worldwide occurrence of diabetes is estimated at about 9% in 2014 [4]. Previous cohort studies suggest that type 2 diabetes was associated with an increased risk of HCC [5]. Findings of a systematic review and meta-analysis of 18 cohort studies reveal that patients with diabetes are associated with two times higher risk of developing HCC than individuals without diabetes [6]. Thus, we need further to explore what factors are associated with HCC risks in patients with diabetes by controlling for known diabetes-related risk factors such as hyperglycemia and metformin use in this line of research.

Serum alanine transaminase (ALT) is a common biomarker of hepatocellular injury. Elevated ALT is related to several liver diseases such as alcoholic liver disease, nonalcoholic fatty liver disease (NAFLD), hepatitis B/C virus infection, drug-induced hepatotoxicity, and autoimmune and cholestatic liver diseases [7]. Several prior cohort studies report elevated ALT is associated with HCC in patients infected with hepatitis B/C virus [8, 9], and a few studies show ALT levels are one of important predictors in predictive models for discriminating HCC patients with hepatitis B/C virus [10]. However, most studies evaluating the relationship between ALT and HCC have focused on patients with B/C virus infection. A recent study examined the relationship between ALT and cancer-specific mortality in diabetic patients but found no such an association existed [11].

Patients with diabetes were been reported to have a higher incidence of liver diseases, including NAFLD, cirrhosis, and acute liver failure [12]. Some studies indicated elevated ALT is more prevalent among diabetic persons [13]. In a community-based study consisting of 11,898 residents, incidence of elevated ALT was 12.5% in type 2 diabetic patients, compared to 7.7% in patients without diabetes [13]. Thus, increased HCC risk in type 2 diabetic patients may be associated with elevated ALT and liver diseases. We conducted a nationwide cohort study of Chinese type 2 diabetic patients with an average follow-up period of 8.17 years to assess whether an elevated ALT level was associated with HCC independently of chronic liver diseases, and to demonstrate its interactive or joint effect with chronic liver diseases on HCC risk in type 2 diabetic patients. Chronic liver diseases considered in this present study were NAFLD, acute liver damage, liver cirrhosis, and hepatitis B and C virus infections.

## RESULTS

Incidence rate of HCC was 2.68 per 1,000 person-years (men: 3.45 and women: 1.98 per 1,000 person-years). Mean age was 60.60 years with a standard deviation (SD) of 11.25 years and mean follow-up period was 8.20 years (SD: 1.86 years). The prevalence of ALT level ≥40 U/L was 22.14% in men and 17.93% in women.

**Figure 1:**
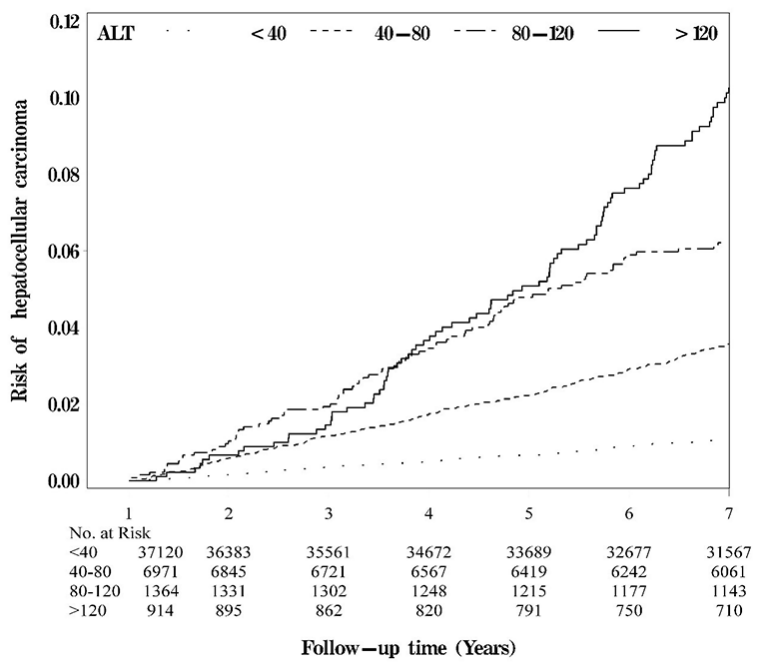
Kaplan-Meier cumulative risk for HCC within subgroups defined by ALT level

Baseline characteristics according to clinical criteria cut-off points of ALT were presented in Table 1. Figure 1 shows Kaplan–Meier cumulative risks of HCC according to subgroups defined by ALT level. Persons with ALT > 120 U/L faced the highest risk, followed by persons with ALT 80–120 and 40–80 U/L, and then persons with ALT≤ 40 U/L had the lowest risk (log-rank test *P* <0.001, Figure 1). Table 2 presents HRs of HCC among patients grouped by ALT levels. Adjusted HRs of HCC in patients with ALT levels of 40–80, 80–120, and > 120 U/L were 3.85 (95% CI 3.34–4.45), 7.05 (5.70–8.71), and 10.85 (8.81–13.36), respectively, after multivariate adjustment (*P* for trend: <0.001), compared with persons exhibiting ALT levels ≤ 40 U/L. When additionally considering BMI and lipid profiles, these three HRs remained similar. When liver diseases were additionally adjusted, the effects of ALT diminished, but remained significant [2.85 (95% CI 2.45–3.31) for 40–80 U/L, 3.80 (3.04–4.76) for 80–120 U/L, and 3.89 (3.08–4.91) for > 120 U/L].

**Table 1:** The comparisons of sociodemographic factors, lifestyle behaviors, diabetes-related variables, drug-related variables, diabetes-related diseases and comorbidity according to ALT level with type 2 diabetes enrolled in the Diabetes Care Management Program, Taiwan (n=46, 369)

Variables	ALT (IU/L)				P value
	≤40 (n=37, 120)	40-80 (n=6, 971)	80-120 (n=1, 364)	>120 (n=914)	
*Sociodemographic factors*					
Male, n (%)^a^	17314 (46.64)	3662 (52.53)	738 (54.11)	522 (57.11)	<0.001
Age (years), mean (SD)^b^	61.31 (11.16)	58.11 (11.06)	56.72 (11.45)	56.59 (11.11)	<0.001
*Lifestyle behaviors*, n (%)^a^					
Smoking	5605 (15.10)	1284 (18.42)	260 (19.06)	215 (23.52)	<0.001
Alcohol drinking	3107 (8.37)	714 (10.24)	154 (11.29)	105 (11.49)	<0.001
*Diabetes-related variables*					
Duration of diabetes (years), mean (SD)^b^	7.21 (8.09)	5.17 (6.71)	4.69 (7.07)	4.57 (6.11)	<0.001
Type of hypoglycemic drug use, n (%)^a^					<0.001
No medication	880 (2.37)	200 (2.87)	29 (2.13)	22 (2.41)	
Metformin only or metformin plus other oral antidiabetic agents	24412 (65.77)	4710 (67.57)	928 (68.04)	558 (61.05)	
Other oral antidiabetic agents	6211 (16.73)	1175 (16.86)	206 (15.10)	159 (17.40)	
Insulin	1069 (2.88)	119 (1.71)	23 (1.69)	27 (2.95)	
Insulin+ oral hypoglycemic drug	4548 (12.25)	767 (11.00)	178 (13.05)	148 (16.19)	
*Drug-related variables*, n (%)^a^					
Hypertension drug treatment	14007 (37.73)	2637 (37.83)	476 (34.90)	281 (30.74)	<0.001
Statin	10284 (27.7)	1852 (26.57)	292 (21.41)	143 (15.65)	<0.001
BMI^a^					<0.001
<18.5	551 (1.48)	56 (0.80)	13 (0.95)	25 (2.74)	
18.5-23.9	11246 (30.3)	1250 (17.93)	255 (18.70)	234 (25.60)	
24-26.9	13143 (35.41)	2256 (32.36)	411 (30.13)	309 (33.81)	
≥27	12180 (32.81)	3409 (48.90)	685 (50.22)	346 (37.86)	
Blood biochemical indexes^a^					
TG (mg/dL)					<0.001
<150	20925 (56.37)	3487 (50.02)	763 (55.94)	558 (61.05)	
≥150	16195 (43.63)	3484 (49.98)	601 (44.06)	356 (38.95)	
FPG (mg/dL)					<0.001
<110	4015 (10.82)	603 (8.65)	129 (9.46)	87 (9.52)	
≥110	33105 (89.18)	6368 (91.35)	1235 (90.54)	827 (90.48)	
HbA1c (%)					<0.001
<7	10911 (29.39)	1700 (24.39)	281 (20.60)	212 (23.19)	
≥7	26209 (70.61)	5271 (75.61)	1083 (79.40)	702 (76.81)	
HDL (mg/dL)					<0.001
≥40(male); 50(female)	17880 (48.17)	3079 (44.17)	637 (46.70)	436 (47.70)	
<40(male); 50(female)	19240 (51.83)	3892 (55.83)	727 (53.30)	478 (52.30)	
LDL (mg/dL)					<0.001
<100	10636 (28.65)	2054 (29.46)	474 (34.75)	389 (42.56)	
≥100	26484 (71.35)	4917 (70.54)	890 (65.25)	525 (57.44)	
Comorbidity^a^					
Acute hepatitis	49 (0.13)	11 (0.16)	5 (0.37)	7 (0.77)	<0.001
Alcoholic liver damage	84 (0.23)	36 (0.52)	5 (0.37)	9 (0.98)	<0.001
Nonalcoholic fatty liver	469 (1.26)	167 (2.40)	41 (3.01)	28 (3.06)	<0.001
Liver cirrhosis	181 (0.49)	104 (1.49)	35 (2.57)	30 (3.28)	<0.001
Cholelithiasis	524 (1.41)	95 (1.36)	23 (1.69)	9 (0.98)	0.56
Alcohol dependence syndrome	47 (0.13)	14 (0.20)	0 (0.00)	3 (0.33)	0.08
Jaundice	14 (0.04)	7 (0.10)	2 (0.15)	3 (0.33)	<0.001
Hepatitis B	213 (0.57)	109 (1.56)	29 (2.13)	21 (2.30)	<0.001
Hepatitis C	70 (0.19)	60 (0.86)	26 (1.91)	40 (4.38)	<0.001
Cholecystitis	76 (0.20)	9 (0.13)	1 (0.07)	0 (0.00)	0.21
Cholangitis	68 (0.18)	10 (0.14)	4 (0.29)	1 (0.11)	0.62
Gastric ulcer	1053 (2.84)	182 (2.61)	37 (2.71)	21 (2.30)	0.58
Duodenal ulcer	718 (1.93)	116 (1.66)	23 (1.69)	25 (2.74)	0.11
Chronic kidney disease	10260 (27.64)	1407 (20.18)	226 (16.57)	155 (16.96)	<0.001
Number of diagnostic testing^b^	0.28 (0.82)	0.29 (0.75)	0.34 (0.90)	0.47 (1.09)	<0.001

a: Differences in categorical variables were tested by the Chi-square test.

b: Differences in continuous variables were tested by the analysis of variance.

ALT: alanine aminotransferase; HCC: hepatocellular carcinoma; LC: liver cirrhosis; ALD: alcoholic liver disease; AFLD: alcoholic fatty liver disease; NAFLD: nonalcoholic fatty liver disease; HBV: hepatitis B virus; HCV: hepatitis C virus.

**Table 2:** Hazard ratios (HRs) of hepatocellular carcinoma according to clinical criteria of baseline ALT level in type 2 diabetic patients enrolled in the NDCMP

Variables	n	Cases	Person-years	IR	Hepatocellular carcinoma (N=1, 018)		
					Model 1	Model 2	Model 3
ALT (IU/L)							
≤40	37120	486	304445.57	1.60	1.00	1.00	1.00
40-80	6971	312	57687.96	5.41	3.85 (3.34, 4.45)^***^	4.00 (3.46, 4.62)^***^	2.85 (2.45, 3.31)^***^
80-120	1364	107	11025.52	9.70	7.05 (5.70, 8.71)^***^	6.99 (5.65, 8.65)^***^	3.80 (3.04, 4.76)^***^
>120	914	113	7140.59	15.83	10.85 (8.81, 13.36)^***^	10.14 (8.22, 12.50)^***^	3.89 (3.08, 4.91)^***^
P for trend∞					<0.001	<0.001	<0.001

Model 1 adjusted for age, sex, duration of type 2 diabetes, smoking, drinking, type of anti-diabetic medication use, anti-hypertension drug treatment and statin use.

Model 2 additionally adjusted for body mass index, triglyceride, fasting plasma glucose, HbA1c, high-density lipoprotein and low-density lipoprotein.

Model 3 additionally adjusted for alcoholic liver damage, nonalcoholic fatty liver, liver cirrhosis, cholelithiasis, alcohol dependence syndrome, jaundice, hepatitis B, hepatitis C, cholecystitis, cholangitis, gastric ulcer, duodenal ulcer, chronic kidney disease and number of image tests.

ALT: alanine aminotransferase; ^***^: p<0.001 for Wald’s test in Cox’s proportional hazards model. ∞: p for trend was from multivariate Cox’s proportional hazards model by treating categorical ALT as an ordinal variable.

Table 3 presents sensitivity analyses by ruling out persons with histories of a stroke, hypoglycemia, coronary artery disease, and HBV, and HCV infection. The association between ALT and HCC remained similar despite the exclusion of persons with the mentioned conditions separately and together. Exclusion of all conditions together (n=39,599) resulted in similar significant HRs for HCC among patients with ALT levels 40–80, 80–120, and > 120 U/L (2.87 [2.43–3.39], 3.92 [3.07-5.02], and 4.53 [3.53–5.81], respectively; *P* for trend<0.001).

**Table 3:** Sensitivity analyses for the association between ALT level and hepatocellular carcinoma in type 2 diabetic patients enrolled in the National Diabetes Care Management Program, Taiwan

Variables	n	Cases	Person-years	IR	Hepatocellular carcinoma
					HR (95%CI)
***Model 1***					
ALT (IU/L)					
≤40	35018	455	289088.10	1.57	1.00
40-80	6702	300	55669.49	5.39	2.88 (2.47, 3.36)^***^
80-120	1310	104	10633.63	9.78	3.96 (3.16, 4.97)^***^
>120	883	112	6917.34	16.19	4.09 (3.23, 5.18)^***^
P for trend∞					<0.001
***Model 2***					
ALT (IU/L)					
≤40	36956	483	303369.80	1.59	1.00
40-80	6962	311	57631.45	5.40	2.85 (2.45, 3.31)^***^
80-120	1364	107	11025.52	9.70	3.80 (3.04, 4.76)^***^
>120	913	113	7131.35	15.85	3.89 (3.08, 4.92)^***^
P for trend∞					<0.001
***Model 3***					
ALT (IU/L)					
≤40	33742	444	278020.00	1.60	1.00
40-80	6423	289	53241.08	5.43	2.84 (2.43, 3.33)^***^
80-120	1275	98	10356.68	9.46	3.79 (3.00, 4.78)^***^
>120	865	112	6752.43	16.59	4.08 (3.22, 5.18)^***^
P for trend∞					<0.001
***Model 4***					
ALT (IU/L)					
≤40	36844	469	302318.20	1.55	1.00
40-80	6803	277	56458.66	4.91	2.83 (2.42, 3.31)^***^
80-120	1310	96	10642.77	9.02	3.72 (2.94, 4.70)^***^
>120	855	102	6714.97	15.19	4.18 (3.28, 5.32)^***^
P for trend∞					<0.001
***Model 5***					
ALT (IU/L)					
≤40	31602	402	262222.60	1.53	1.00
40-80	6034	247	50341.02	4.91	2.87 (2.43, 3.39)^***^
80-120	1180	87	9660.26	9.01	3.92 (3.07, 5.02)^***^
>120	783	100	6165.69	16.22	4.53 (3.53, 5.81)^***^
P for trend∞					<0.001

Model 1 excludes patients with stroke (n=2,456); Model 2 excludes patients with hypoglycemia (n=174); Model 3 excludes patients with coronary artery disease (n=4,064); Model 4 excludes patients with hepatitis B or hepatitis C (n=557); Model 5 excludes patients with stroke, hypoglycemia, coronary artery disease, hepatitis B or hepatitis C (n=6,770).

Adjustment for age, sex, duration of T2DM, smoking, drinking, type of diabetes treatment, anti-hypertension drug treatment, statin use, body mass index, triglyceride, fasting plasma glucose, HbA1c, high-density lipoprotein, low-density lipoprotein, alcoholic liver damage, nonalcoholic fatty liver, liver cirrhosis, cholelithiasis, alcohol dependence syndrome, jaundice, hepatitis B (except model 4 and 5), hepatitis C (except model 4 and 5), cholecystitis, cholangitis, gastric ulcer, duodenal ulcer chronic kidney disease and number of image tests.

ALT: alanine aminotransferase; ^***^: p<0.001 for Wald’s test in Cox’s proportional hazards model. ∞: p for trend was from multivariate Cox’s proportional hazards model by treating categorical ALT as an ordinal variable.

**Figure 2:**
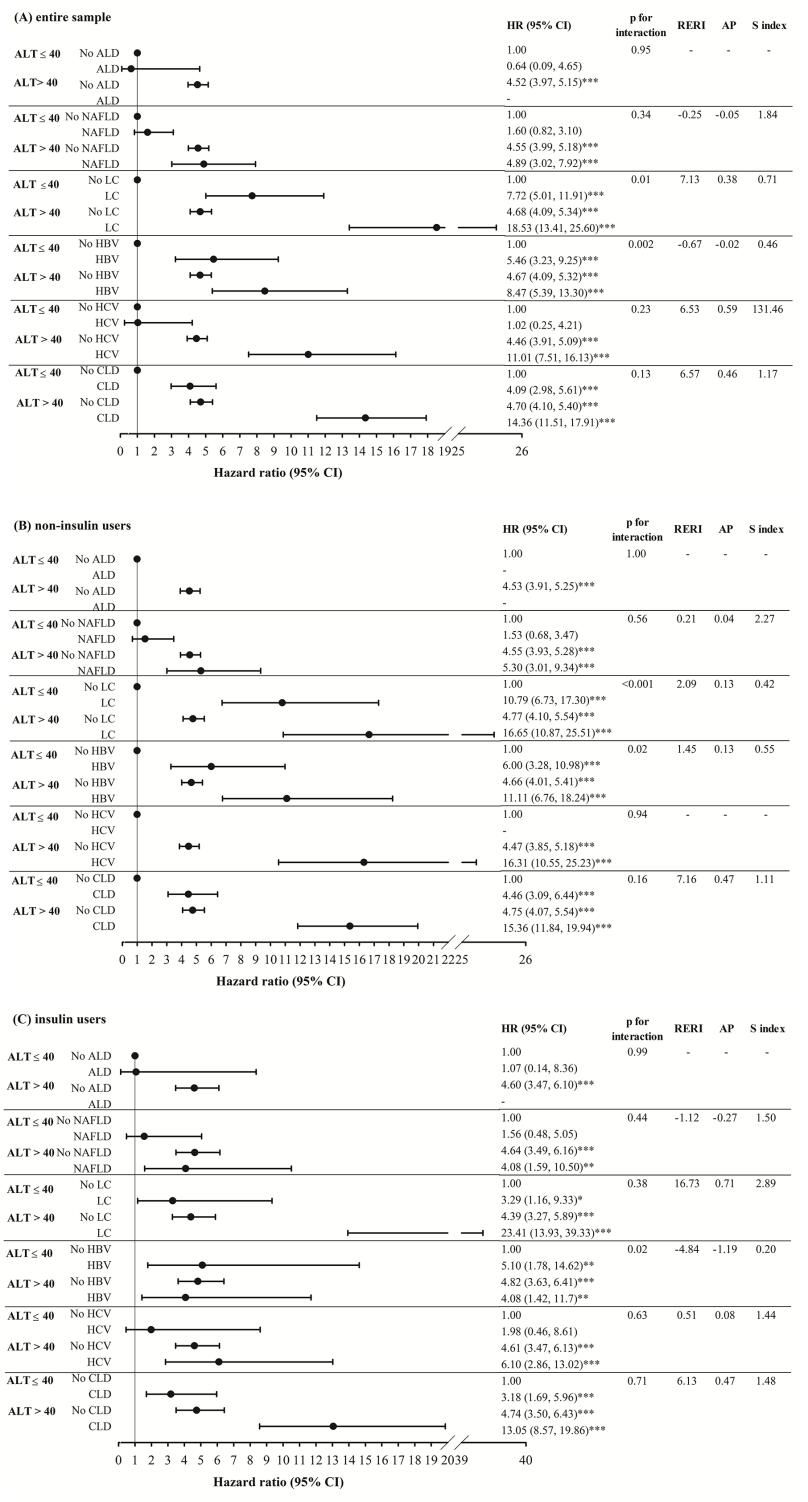
The adjusted HR of HCC for the effects of ALT>40 and alcoholic liver damage, nonalcoholic fatty liver disease, liver cirrhosis, hepatitis B virus infection, hepatitis C virus infection, and any one of these chronic liver diseases for the entire sample, and stratified by insulin use ALD: alcoholic liver damage; NAFLD: nonalcoholic fatty liver disease; LC: liver cirrhosis; HBV: hepatitis B virus infection; HCV: hepatitis C virus infection; CLD: chronic liver diseases.

Figure 2 demonstrates adjusted HRs of HCC for joint effects of ALT > 40 U/L and ALD, NAFLD, liver cirrhosis, HBV and HCV infection, and any one of these CLD for entire sample, and stratified by insulin use. We observed more significant HRs of HCC for patients with a level of ALT > 40 U/L with NAFLD, liver cirrhosis, HBV and HCV infection, and any one of these CLDs than those of patients with a level of ALT ≤ 40 U/L and no counterpart comorbidity (4.89, 3.02–7.92; 18.53, 13.41–25.60; 8.47 5.39–13.30; 11.01, 7.51-16.13, and 14.36, 11.51–17.91, respectively). Significant interactions were observed between ALT level with liver cirrhosis, and HBV (p for interaction terms =0.01 and 0.002). Stratified analysis was performed according to insulin use. Similar significant joint effects of ALT > 40 U/L with NAFLD, liver cirrhosis, HBV and HCV infection, and any one of these CLDs were observed in insulin, and non-insulin users. Main effects of ALT > 40 U/L were all significant across subgroups of various chronic liver diseases with a narrow 95% CIs, and remained similar in the entire sample, non-insulin users, and insulin users. In general, PERI, AP, and S-index indicated that interaction of ALT > 40 U/L with liver cirrhosis was positive, but interaction of ALT > 40 U/L with HBV was negative.

## DISCUSSION

We investigated the relationship between ALT level and HCC risk among Chinese type 2 diabetic patients in Taiwan NDCMP. This nationwide cohort study had 46,396 type 2 diabetic patients, aged 30 years and over at baseline. This study demonstrated an independent association between ALT level and HCC risk. Findings of sensitivity analysis illustrated this independent association between ALT level and HCC risk was similar after ruling out persons with stroke, hypoglycemia, other cancers, and HBV and HCV infection at baseline. Our consistent findings of sensitivity analysis provided evidence to support robustness of our study results. We identified significant effect modifications of ALT level with liver cirrhosis and HBV. Joint effects of ALT level and chronic conditions were demonstrated by significant combined associations of ALT > 40 U/L and NAFLD, liver cirrhosis, HBV and HCV infection, or any one of CLDs with HCC risk.

ALT is a transaminase enzyme, one of intracellular hepatic enzymes leaking into circulation, and served as part of a diagnostic evaluation of hepatocellular injury. Although an elevated ALT level was uncommon in general population, obese individuals and type 2 diabetic patients were reported to have elevated ALT levels, which can be accounted for by increased body mass index [14]. We observed synergistic effects of ALT and liver cirrhosis on HCC risk. The possible biological mechanisms may explain this effect modification included insulin resistance and inflammation, which were two possible biological mechanisms for diabetes as a risk factor of cancer. The most common cause of a mild elevation of ALT in type 2 diabetic patients was NAFLD [15]. The incidence of NAFLD in diabetes was high and was 100% in patients with obesity. NAFLD was the hepatic manifestation of insulin resistance syndrome [16] with a spectrum of liver disease from fatty infiltration of liver to nonalcoholic steatohepatitis (NASH), consisting of steatosis with inflammation, necrosis, and fibrosis, which further lead to cirrhosis. Insulin resistance was central abnormality in pathogenesis of steatosis.

Prior studies reported the association between ALT and HCC in patients with a high risk of HCC such as patients with cirrhosis and hepatitis B/C virus infection [8–10]. Our study reported an elevated ALT level increased HCC risk in Chinese type 2 diabetic patients, indicating significant interactions for ALT ≥ 40 IU/L with liver cirrhosis and HBV on HCC risk as well as joint associations of ALT ≥ 40 U/L and chronic liver diseases. In a recent study conducted by Williams et al. [11], they have failed to find an association between ALT levels and cancer mortality in patients with type 2 diabetes. The possible reasons that can explain the different findings are the smaller sample size in Williams’s study (n=9,795 vs. n=46,369) and the shorter follow-up period (5 years vs. 8 years). Our findings regarding joint effect allow us to distinguish the effects of elevated ALT alone, and cumulative effects of elevated ALT and chronic liver diseases such as hepatitis B/C virus infection, cirrhosis, and NAFLD. The consistent findings that elevated ALT alone may imply that evaluated ALT independently leading to HCC, irrespective of the other known pro-oncogenic effects of hepatitis B/C virus infection, and cirrhosis.

The clinical implication of our study findings is that ALT management has to take chronic liver diseases into account. Although elevated ALT is not a life-threatening condition, high ALT does warrant prompt medical attention. Elevated ALT indicates damage to liver caused by life-threatening diseases or infections and mainly reflect underlying liver injury, mainly from NAFLD, HBV, HCV chronic hepatitis and cirrhosis. If high ALT with known cause is identified, the treatments for both chronic liver diseases and high ALT should be applied. Chronic liver disease medications included ursodeoxycholic acid for slowing the progression of primary biliary cirrhosis, antiviral medications for reduction of liver enzymes elevated by hepatitis C, etc. If high ALT without known cause was identified, then medication and diet modification should be considered including corticosteroids and pentoxifylline for liver inflammation reduction, diuretics for removing excess fluid from the body, avoiding intake of salt and alcohol-drinking, and a low-protein diet for reduction of the risk of toxins building up in the body, and weight loss.

Prior studies regarding the association of ALT with HCC in type 2 diabetic patients are limited, and most of them had been conducted in general population [17, 18] or on individuals with cirrhosis [19], HBV [8, 9, 20], or HCV infections [21] who are at high risk. Two studies that developed HCC prediction models in Asian general population were conducted [17, 18]. One study revealed that ALT levels was a significant predictor of HCC risk in Chinese patients with an unknown or HBV- or HCV-negative infection status [17]. On the contrary, ALT level wasn’t incorporated into HCC prediction model in a Japanese population [18]. Among studies conducted in patients with a high risk of HCC, no study has reported ALT level to be a significant predictor in patients with cirrhosis or HCV infection [19, 21]. On the contrary, ALT level was a significant predictor among studies that explored factors associated with HCC, or developing scoring systems to predict HCC among patients with chronic HBV infection [8, 9, 20].

Several studies explored predictors for HCC in type 2 diabetic patients. In a study with a sampling scheme based on insulin use status, HbA1c, was the key predictor for HCC, and ALT level was not considered in the potential factors [22]. Another cohort study focused on the effect modification of HBV infection with lipid profiles and medication use on liver cancer, but it did not consider ALT level [23]. Our study has focused on the association between ALT level and HCC risk, and has directed our research effort on the primary objective to create a strong basis for interpreting the study results. Hence, our study provides credible findings showing joint effect of ALT level and chronic liver diseases on HCC risk in Chinese type 2 diabetic patients.

This study has several strengths. First, this study had a nationwide cohort with a large sample size to assess whether elevated ALT levels increased HCC risk in Chinese type 2 diabetic patients enrolled in NDCMP. Second, baseline information was collected before subsequent diagnosis of HCC. This process prevents recall bias inherent in case-control studies with exposure collected after HCC diagnosis. Finally, we considered many factors, including lifestyle habits, anti-diabetes treatment, comorbidity, and biomarkers of FPG, lipid profiles and HbA1c in multivariable models.

However, several limitations are noted. First, our datasets do not contain information of aspartate aminotransferase (AST) and alpha-fetoprotein (AFP) markers, which had been demonstrated to be associated with HCC in general population. Thus, we cannot adjust for the confounding effects of these two biomarkers. In addition, the database does not contain information on leisure-time physical activity, and dietary habits, which may also be risk factors for liver cancer. Future studies linking administrative data, personal information is warranted. Second, a potential measurement error because of undiagnosed or misdiagnosed liver cancer cases may exist. However, the likelihood of overdiagnosis would be small because of audit system of clinic and hospital records. NHI program regularly conducts expert reviews of patient charts to confirm the validity of randomly selected claims from all hospitals. False or inconsistent reports will incur severe penalties. In addition, HCC cases were defined as patients with at least three ambulatory claims or at least one inpatient care claim for HCC to improve true positive rates. However, errors arising from miscoding and misclassification may be random, which would result in underestimation of the effect if the association between ALT and HCC exists. This implied that true effect would be stronger, which would be a lesser threat to the validity of our findings. Third, it is very likely to underdiagnose NADLD, HBV, HCV infection and cirrhosis because the tests for HBV and HCV infection, cirrhosis, and NAFLD were not regular check-up items for diabetes care, the tests weren’t offered for patients with no probable indication or symptoms. Thus those who didn’t have probable symptoms weren’t seek for health care and won’t be identified. If the un-diagnosis is random according ALT level, the impact of the un-diagnosis would result in underestimation for the independent effects of ALT level and these chronic liver diseases as well as their interactions. If the un-diagnosis is not random, it is likely that patients in the lower levels of ALT had a higher likelihood of un-diagnosis for these chronic liver diseases. The impact of the un-diagnosis would result in underestimating the independent and interactive effects of ALT level and these chronic liver diseases. Because the potential un-diagnosis bias results in the effect that may be toward the null, a lesser threat to validity of the findings. Finally, potential selection bias might be possible because of differential characteristics between type 2 diabetic patients who enrolled and did not enroll in NDCMP. To evaluate the potential selection bias, we made comparisons of age and gender distributions between our study subjects and type 2 diabetes population using NHIRD dataset, and we found similar distributions (difference in mean age was 1.5 years and in female proportion was less than 1%). Non-differential distributions in age and gender imply this kind of selection error might be random; thus, biased results on the effects may be null and would be a lesser threat to validity.

In conclusion, our study demonstrates an elevated ALT level increased HCC risk in Chinese type 2 diabetic patients. This result indicates significant interactions were observed for ALT ≥ 40 IU/L with liver cirrhosis and HBV on HCC risk. Our study provides new insights for health professionals to target patients with diabetes who are at higher risks of HCC. Elevated ALT level should warrant medical attention. Diabetes care should provide lifestyle or treatment interventions to manage ALT level, liver cirrhosis and HBV for reducing HCC burden.

## MATERIALS AND METHODS

### Study population

**Figure 3:**
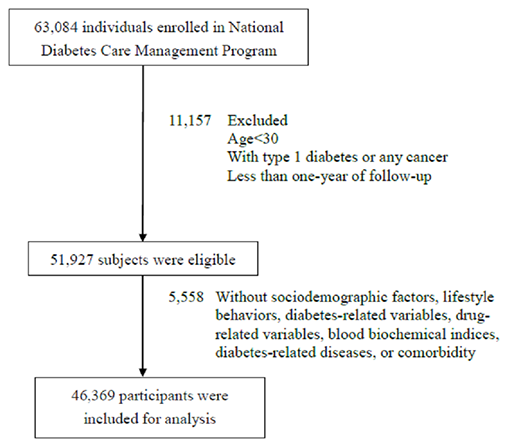
Flowchart of recruitment procedures for the current study

We carried out a nationwide retrospective cohort study, Taiwan Diabetes Study, among enrollees in National Diabetes Care Management Program (NDCMP), founded by Ministry of Health and Welfare of Taiwan in 2001 for enhancing the quality of diabetes care. NDCMP provides additional financial incentives for care providers to have their eligible patients enrolled in this program and to have continuing clinical education and training programs for certification. Taiwan Diabetes Study, a nationwide cohort study, consisted of 63,084 ethnic Chinese type 2 diabetic patients registered in NDCMP in 2002-2004. We used date of entry into NDCMP as index date. Patients with a clinically confirmed diagnosis of diabetes mellitus based on American Diabetes Association criteria (International Classification of Diseases, ninth revision, Clinical Modification (ICD-9-CM) diagnosis code 250) were invited by their primary care providers to enroll in this program. We ruled out persons who had type 1 diabetes (ICD-9-CM; code 250.x1/x3) and gestational diabetes, patients aged under 30 years, diagnosed with any cancers at baseline, and less than one year of follow-up (n=11,157). Enrollees with missing data on sociodemographic factors, lifestyle behaviors, blood biomarkers, and medication use (n = 5,558) were also excluded from analysis. Finally, 46,369 enrollees were qualified (22,236 men and 24,133 women) in the analysis (Figure 3). This study was approved by Ethical Review Board of China Medical University Hospital. Informed consent of study participants was not required because dataset used in this study consists of de-identified secondary data released for research purposes.

### Data sources for baseline and follow-up assessments

We used datasets of admission for inpatient care and ambulatory care visits during 2002–2011. Every person in Taiwan has a unique personal identification number (PIN). To ensure patients’ security and privacy, NHIRD provided data with patient identities being encrypted. Researchers can interlink all NHI datasets through encrypted PIN. Data consists of information on sociodemographic variables, date, treatments and source of diagnosis for ambulatory care and inpatient admission. ICD-9-CM codes were used to identify individual comorbidity status. Proportion of enrollees leaving from NHI program is fairly low because of comprehensive coverage of this program. Thus, loss follow-up bias is negligible.

NDCMP is a multidisciplinary case management program run by Ministry of Health and Welfare of Taiwan since 2001. This program provided diet, physical activity, and enhanced self-care education, annual diabetes-specific physical examinations and laboratory tests, as well as continuity of care to decrease diabetes-related complications. On the date of entry into the NDCMP, enrollees underwent a physical check-up, consisting of comprehensive assessment of disease and complication status, along with a series of blood tests, urine tests, and body measurements. Enrollees had to complete a standardized and computerized questionnaire by a case management nurse to record previous or current disease status, medication, and lifestyle habits. Smoking and alcohol drinking status were according to patients’ self-report. The smoking group consisted of patients who were current or past smokers and non-smoking group included those who had never smoked. Blood was extracted from an antecubital vein in the morning, after a 12-hour overnight fast, and sent for analysis within four hours after collection. To diagnose a susceptible individual for acute HBV in clinical settings in Taiwan, an HbsAg test is ordered. The test for serum HBV DNA is optional. If the test result for HbsAg is positive, it would be compatible with acute HBV infection. The HBsAg test has to be repeated in six months. If the test result is positive for more than six months, it is defined as chronic HBV based on the definition of clinical guideline for chronic HBV infection that the continued presence of HBsAg in the blood for longer than six months. For HCV infection, an individual with positive anti-HCV antibodies and detectable HCV RNA is defined as hepatitis C infection. In addition, none of these patients have history of hemorrhage from esophageal varices or ascites, or hepatic encephalopathy. Whether an individual is diagnosed as having cirrhosis is determined by peritoneoscopy, biopsy or both, and by clinical symptoms with ultrasonographic findings. An individual with a typical irregular-surfaced liver with coarse internal architecture by the ultrasonography in addition to overt ascites or esophageal varices demonstrated by fiberscopic examination is defined as cirrhosis. NAFLD includes a spectrum of liver disease ranging from simple steatosis (non-alcoholic fatty liver [NAFL]) to non-alcoholic steatohepatitis (NASH). For diagnosis of NAFLD, it is required there is evidence of steatosis either by imaging or histology and no secondary causes of steatosis, such as viral hepatitis, increased alcohol consumption, use of steroid medications or other causes. The diagnosis of steatosis is based on a liver biopsy or imaging techniques such as ultrasound. A positive test result for NAFL if a liver biopsy with ≥5–10% of hepatocytes exhibiting macroscopic steatosis or the semi-quantitative image of ultrasound indicating any degree of steatosis alone or steatosis with lobular inflammation but without ballooning. NASH is only diagnosed by liver biopsy with the presence of ballooning injury.

### Outcome ascertainment

Primary outcome was HCC, determined by ambulatory and inpatient care data in NHIRD. HCC incident cases were ascertained by codes (155 for HCC) of ICD-9-CM. All HCC cases met at least one of the following criteria to enhance its true positive rate: at least three ambulatory claims or at least one inpatient care claim. A total of 1,018 patients with HCC incidence were identified from this cohort. Follow-up time began with index date and ended with a newly diagnosed HCC, death, withdrawal from the insurance program, or end of follow-up on December 31, 2011. The diagnosis of HCC in Taiwan is based on clinical, imaging, and histopathological findings. All patients are asked about their medical history to check for risk factors and symptoms, and are examined for signs of liver cancer. If symptoms or the physical exam results suggest patients might have liver cancer, imaging tests are performed during the preoperative period such as ultrasonography, CT scan, or abdominal MRI. Lab tests such as alpha-fetoprotein and liver function are ordered to determine what might have caused the liver cancer, and how well the liver function, which can affect types of treatments. In some cases, a biopsy is needed. For those who have surgery, all specimens are obtained.

The validity of the cancer diagnosis in the NHIRD has been reported previously [24] using all newly diagnosed people with cancer between January 1, 2001 and December 31, 2012. The cancer cases from the National Cancer Registry (NCR) in Taiwan had been treated as true cases. The estimates of the validity of cancer diagnoses in the NHI database by sensitivity, specificity, positive (PPV) and negative (NPV) predictive values were 80.07%, 99.99%, 90.39%, and 99.99%, respectively. This study’s findings provide the evidence to support NHI database is a valid source for cancer epidemiology study. The data source for true cases was NCR, which is implemented by the Ministry of Health and Welfare and is a compulsory system that asks hospitals caring cancer patients to provide valid personal, clinical, pathology, laboratory, and imaging data. The Taiwan NCR’s data quality and validity of diagnostic criteria methods followed the definitions proposed by [25]. It has been reported that the proportions of death certificate-only and morphological verification cases in Taiwan [26] were comparable with cancer registries of Iceland [27], Norway [28], and Singapore [29].

### Covariates

Data on comorbid conditions were extracted from NHIRD for a 24-month period prior to index date by using outpatient and inpatient claim data. Instead of a 12-month period, we specified a 24-month period because a few of these comorbid conditions are not common. To ascertain that we do not miss patients’ diagnosis, we required a longer period. For the number of image tests, we extracted outpatient and inpatient claim data from NHIRD for a 24-month period after index date.

Data on medication uses prescribed for diseases were calculated for 12-month period prior to cohort entry. Outpatient prescriptions within one year of index date were used to define their anti-diabetes or statin medication use. A patient was a user of anti-diabetes or statin if his/her number of prescription days was greater than 90 days. A patient may have more than one type of anti-diabetes medication use if she/he had more than one medication use. We classified anti-diabetes medications into: no medication, sulfonylurea monotherapy or sulfonylurea plus oral anti-diabetes drug (OAD) monotherapy other than metformin or sulfonylurea (OAD-other), metformin monotherapy or metformin plus OAD-other combination, metformin plus sulfonylurea combination, OAD-other monotherapy or OAD-other combination, insulin monotherapy, and insulin plus one or more OAD.

### Statistical analysis

Baseline measurement of ALT was determined based on datasets of electronic lab records. ALT level at baseline was grouped into four categories according to clinical criteria: ≤40, 40–80, 80–120, and > 120 U/L. Cox proportional hazards models were utilized to assess the association between ALT level and HCC risk for multivariable adjustment. Hazard ratios (HRs) and 95% confidence intervals (CIs) were estimated under three multivariable models. The first multivariable model adjusted for age, gender, smoking status, alcohol drinking status, diabetes duration, statin use, and type of hypoglycemic drug and anti-hypertension drug treatment. The second one additionally adjusted for blood biomarkers including HbA1c, fasting plasma glucose (FPG), high-density lipoprotein (HDL), low-density lipoprotein (LDL), triglyceride (TG), and body mass index (BMI). The third one further included time-varying comorbid conditions. Proportional hazards assumption was verified by the graph of log (−log(survival)) versus log of survival time graph and by statistical significance test of a covariate that allowed time-varying ALT. No significant violation was found. To test the trend of ALT, categorical ALT had been treated as an ordinal variable by coding the four categories of ≤40, 40–80, 80–120, and > 120 U/L from 1 to 4. To account for the effects of other variables, this ordinal variable was entered into the multivariate Cox’s proportional hazards model along with the other variables and p value for this ordinal variable was reported as p for trend. Three dummy indicators were created to assess joint effect of ALT and each chronic liver disease. Using individuals with ALT level ≤ 40 U/L and without chronic liver disease as reference, these three dummy indicators estimated independent effects of ALT level > 40 U/L only, chronic liver disease only, and both ALT level > 40 U/L and chronic liver disease. Interactions of ALT level > 40 U/L with age, gender, ALD, NAFLD, liver cirrhosis, HBV and HCV infection, and any one of chronic liver diseases (CLD) were examined by including their product terms into the full model, and its significance was tested by likelihood ratio test. Moreover, proportion attributable to interaction (AP), relative excess risk due to interaction (RERI), and synergy index (S index) were derived. A zero value of PERI or AP indicates no interaction, a positive value of PERI or AP indicates positive interaction, and a negative value of PERI or AP indicates negative interaction. A value of one for S index indicates no interaction, a value of greater than one indicates positive interaction, and a value of less than one indicates negative interaction.

Sensitivity analyses were performed to examine robustness of our findings. The main analyses were repeated by excluding participants with stroke, hypoglycemia, coronary artery disease and HBV and HCV infection separately and together. SAS version 9.4 (SAS Institute Inc., Cary, NC) was used for all analyses. A two-sided level of significance was specified at 0.05.
